# Lung Targeted Lipopolymeric Microspheres of Dexamethasone for the Treatment of ARDS

**DOI:** 10.3390/pharmaceutics13091347

**Published:** 2021-08-27

**Authors:** Sabna Kotta, Hibah Mubarak Aldawsari, Shaimaa M. Badr-Eldin, Lenah S. Binmahfouz, Rana Bakur Bakhaidar, Nagaraja Sreeharsha, Anroop B. Nair, Chandramouli Ramnarayanan

**Affiliations:** 1 Department of Pharmaceutics, Faculty of Pharmacy, King Abdulaziz University, Jeddah 21589, Saudi Arabia; haldosari@kau.edu.sa (H.M.A.); smbali@kau.edu.sa (S.M.B.-E.); rbakhaidar@kau.edu.sa (R.B.B.); 2 Center of Excellence for Drug Research and Pharmaceutical Industries, King Abdulaziz University, Jeddah 21589, Saudi Arabia; 3 Department of Pharmacology and Toxicology, Faculty of Pharmacy, King Abdulaziz University, Jeddah 21589, Saudi Arabia; lbinmahfouz@kau.edu.sa; 4 Department of Pharmaceutical Sciences, College of Clinical Pharmacy, King Faisal University, Al-Ahsa 31982, Saudi Arabia; sharsha@kfu.edu.sa; 5 Department of Pharmaceutics, Vidya Siri College of Pharmacy, Off Sarjapura Road, Bangalore 560035, India; 6 Department of Pharmaceutical Chemistry, Vidya Siri College of Pharmacy, Off Sarjapura Road, Bangalore 560035, India; pharmwhiz@gmail.com

**Keywords:** lung inflammation, ARDS, microspheres, lung targeting, dexamethasone

## Abstract

Acute respiratory distress syndrome (ARDS), a catastrophic illness of multifactorial etiology, involves a rapid upsurge in inflammatory cytokines that leads to hypoxemic respiratory failure. Dexamethasone, a synthetic corticosteroid, mitigates the glucocorticoid-receptor-mediated inflammation and accelerates tissue homeostasis towards disease resolution. To minimize non-target organ side effects arising from frequent and chronic use of dexamethasone, we designed biodegradable, lung-targeted microspheres with sustained release profiles. Dexamethasone-loaded lipopolymeric microspheres of PLGA (Poly Lactic-co-Glycolic Acid) and DPPC (Dipalmitoylphosphatidylcholine) stabilized with vitamin E TPGS (D-α-tocopheryl polyethylene glycol succinate) were prepared by a single emulsion technique that had a mean diameter of 8.83 ± 0.32 μm and were spherical in shape as revealed from electron microscopy imaging. Pharmacokinetic and biodistribution patterns studied in the lungs, liver, and spleen of Wistar rats showed high selectivity and targeting efficiency for the lung tissue (*r_e_* 13.98). As a proof-of-concept, in vivo efficacy of the microspheres was tested in the lipopolysaccharide-induced ARDS model in rats. Inflammation markers such as IL-1β, IL-6, and TNF-α, quantified in the bronchoalveolar lavage fluid indicated major improvement in rats treated with dexamethasone microspheres by intravenous route. Additionally, the microspheres substantially inhibited the protein infiltration, neutrophil accumulation and lipid peroxidation in the lungs of ARDS bearing rats, suggesting a reduction in oxidative stress. Histopathology showed decreased damage to the pulmonary tissue upon treatment with the dexamethasone-loaded microspheres. The multipronged formulation technology approach can thus serve as a potential treatment modality for reducing lung inflammation in ARDS. An improved therapeutic profile would help to reduce the dose, dosing frequency and, eventually, the toxicity.

## 1. Introduction

In the current situation of the COVID-19 pandemic, the world has witnessed a huge loss of life due to respiratory complications worldwide. The COVID-19 pandemic has once again reinforced the fact that respiratory illness often accelerates morbidity thus making the health conditions difficult to manage [1,2]. Additionally, a multitude of respiratory diseases, such as pulmonary hypertension, acute respiratory distress syndrome (ARDS), chronic obstructive pulmonary disease, asthma, idiopathic pulmonary fibrosis, lung cancer, and infectious lung diseases like tuberculosis also contribute to major global health burdens involving all the age groups and greatly affect the global economy [3]. ARDS is typically observed in critically ill or patients with sepsis, severe pneumonia, coronavirus disease, or significant head or chest injuries [4]. Acute lung injury causes disruption of the lung endothelial and epithelial barriers. As a consequence, the lung’s mechanics change and gas exchange are compromised. Severe shortness of breath is the main symptom of ARDS which usually develops within a few hours and lasts for several days, thus precipitating the injury that eventually necessitates mechanical ventilation. Despite some improvements, mortality remains high at 30–40% in most studies [5]. There are currently no specific effective therapies for ARDS and thus there is a great need for novel therapeutic approaches. Several drugs, including nitric oxide, ketoconazole, heparin, and ibuprofen, have been investigated, but none have been shown to improve patient outcomes [6]. Early administration of dexamethasone could reduce the duration of mechanical ventilation and mortality in patients with moderate-to-severe ARDS [6,7,8]. Dexamethasone, a synthetic corticosteroid, downregulates glucocorticoid-receptor-mediated inflammatory cascade, mitigating inflammation and accelerating tissue homeostasis towards disease resolution [9]. However, chronic use of dexamethasone even at low doses is associated with significant side effects [10,11,12]. Thus, there is a need for the formulation with lung-targeted delivery potential for the treatment of ARDS. Although there are several reports on nanoparticles for lung delivery [13,14], none of the formulations, except Abraxane, could achieve commercial success. The major drawback of nanotechnology is the difficulty in technology transfer, complexity in commercial scale-up, and toxicity concerns. In contrast to nanoparticles, microspheres offer a well-established platform technology for easy commercialization and effective delivery by oral, parenteral as well as nasal routes [15,16]. The methodology applies to a wide range of therapeutics with diverse physicochemical properties like small antibiotic molecules and peptides, and proteins [17]. Huo et al. [18] showed that cisplatin-loaded biodegradable PLGA microspheres could achieve successful accumulation of particles in lungs after parenteral administration along with a sustained release for effective long-term therapy to reduce the frequency of dosing. The potential of microspheres to effectively deliver carboplatin to the lungs after intravenous administration was also demonstrated [19]. Recently, Sreeharsha et al. [20] successfully developed salbutamol-loaded PLGA-PEG microspheres for the treatment of asthma. Qu et al. [21] compared two different techniques of microspheres preparation namely, emulsion cross-linking and spray-drying. Cefquinome was loaded into biocompatible gelatin microspheres for lung targeting that showed significantly higher drug loading and encapsulation efficiency. Ung et al. [22] explored insulin microspheres bypassing the extra-thoracic deposition and achieving maximum lung deposition. Razuc et al. [23] successfully formulated ciprofloxacin microspheres using simple and green spray drying technology with high yield and excellent powder flow characteristics. In view of these findings, encapsulating dexamethasone in microspheres seems to be a potential approach for targeting it to lungs.

Therefore, the present study focuses on the development of dexamethasone-loaded lipopolymeric microspheres comprising 1,2-dipalmitoyl-sn-glycero-3-phosphocholine (DPPC) and Poly (lactic-co-glycolic acid) (PLGA) for targeted lung delivery. Being the main constituent of endogenous pulmonary surfactant and ability to reach deep alveoli due to surface activity, we used DPPC in the formulation. Emulsification was facilitated by the use of Vit E TPGS. After successfully achieving physicochemical properties in terms of morphology, size, encapsulation efficiency, and in vitro release profile, a proof-of-concept was provided using in vivo studies in laboratory animals. The pharmacokinetics, biodistribution and lung-targeting efficiency of dexamethasone microspheres in Wistar rats after intravenous route of administration supported the technology hypothesis. Further, the capability of lowering the inflammation markers and reducing the oxidative stress in lipopolysaccharide (LPS) induced ARDS model in rats strengthened the efficacy and applicability potential of the developed formulation for targeted lung delivery.

## 2. Materials and Methods

### 2.1. Materials

Dexamethasone and DPPC were purchased from Cayman Chemicals, Ann Arbor, MI, USA and Avanti Polar Lipids, Alabaster, AL, USA. PLGA, ester terminated lactide: glycolide 75:25, ΜW- 76,000–115,000), Vitamin E TPGS (D-α-tocopheryl polyethylene glycol-1000 succinate), and LPS from *Escherichia coli* O111:B4 were purchased from Sigma-Aldrich (St. Louis, MO, USA). Potassium phosphate, sodium chloride, potassium chloride, calcium chloride, formaldehyde, dichloromethane, phosphoric acid, methanol, and acetonitrile were purchased from Merck Chemicals, India. Distilled water was used in all experimental procedures. Elisa kits were purchased from RayBiotech, Peachtree Corners, GA, USA.

### 2.2. Methods

#### 2.2.1. Preparation of Dexamethasone Microspheres and Estimation of Loading and Encapsulation Efficiency

Dexamethasone-loaded microspheres were prepared by a single emulsion technique [24]. The lipid phase consisted of PLGA:DPPC (4:1) and the drug to lipid ratio was 2:8. The lipid phase was emulsified with an aqueous phase containing vitamin E TPGS (0.25% *w*/*v*) at 10,000 rpm, for 20 min using a high-speed homogenizer (T25, Ultra-Turrax, Ika^®^, Staufen, Germany) at ambient temperature. The resultant homogeneous emulsion was stirred at 800 rpm under magnetic stirring for solvent evaporation. Further, the microspheres were separated by centrifugation at 10,000 rpm at 4 °C, washed with distilled water, and lyophilized. The dried microspheres were stored in a desiccator until further analysis. Loading and encapsulation efficiency were calculated using dexamethasone content estimated using the RP-HPLC method as mentioned below.

#### 2.2.2. Determination of Particle Size, Distribution and Imaging Using Electron Microscopy

For the determination of particle size, the microspheres were suspended in normal saline and the size distribution was analyzed using laser diffraction analysis (Malvern Mastersizer 300, Malvern, UK) [25]. The particle size was expressed as volume-weighted mean diameter in micrometers [26]. The width of the size distribution was calculated and expressed as a polydispersity index. Six runs of size measurement were performed at 25 °C and the mean particle size was calculated.

For scanning electron microscopy (SEM), the microspheres were placed on carbon conductive adhesive tape mounted on the specimen stub. The mounted sample was frozen at −190 °C in liquid nitrogen and transferred to the preparation chamber, maintained at −130 °C, and sublimed at −90 °C for 10 min, followed by coating with platinum [27]. It was then transferred to the SEM chamber for viewing at −150 °C with an accelerating voltage of 5.0 kV (JSM-7600F field emission gun SEM equipped with Cryo unit (PP3000T) by Quorum). For transmission electron microscopy (TEM), the microspheres were mounted on a carbon-coated formvar grid, stained with neutralized 2% phosphotungstic acid and dried before imaging [28].

#### 2.2.3. In Vitro Release of Dexamethasone

Dexamethasone-loaded microspheres (10 mg) were transferred to a Float-A-Lyzer (G2, Spectrum, Repligen, MA, USA) and suspended in 1 mL release media, phosphate buffer saline pH 7.4 at 37 °C (*n* = 6) [29]. The dialyzer was then introduced into covered beakers containing 500 mL release media and stirred at 100 rpm on a magnetic stirrer [30]. Dexamethasone release was analyzed at predetermined time intervals by withdrawing 0.5 mL of release media. The volume of release media was maintained at 50 mL by replacing equally of release media immediately after sampling. Dexamethasone content was determined by using the validated RP-HPLC method.

#### 2.2.4. Analysis of Dexamethasone by HPLC

Dexamethasone content was estimated using the validated RP-HPLC method. All test samples were diluted suitably with mobile phase and the chromatographic separation was performed using an isocratic elution. The mobile phase consisted of a mixture of potassium phosphate buffer (10 mM, pH adjusted to 3.0 using dilute orthophosphoric acid) and acetonitrile (60:40) and delivered at a flow rate of 1 mL/min. The HPLC system consisted of a pump (Jasco PU-2080 Plus, Intelligent HPLC pump, Tokyo, Japan) connected to Detector (Jasco 2075, Intelligent UV–Vis detector, Tokyo, Japan). The separation was carried out at 20 °C, on a reversed-phase C18 column (Qualisil^®^ BDS, 250 × 4.6 mm, 5 μm particle size, Qualisil, Agilent technologies, Mumbai, India). An injection volume of 20 μL was used. Detections were carried out at 242 nm. The method was validated for accuracy, precision, specificity, and solution stability.

#### 2.2.5. Pharmacokinetic, Biodistribution and Targeting Efficiency

Eight-week-old, healthy, laboratory-bred, Wistar rats of either sex, weighing 200 ± 20 g were maintained at a temperature of 25 ± 2 °C and a 12 h natural light period. Commercial pellet diet and tap water were provided ad libitum. The experiments were conducted at CPCSEA (Committee for the Purpose of Control and Supervision of Experiment on Animals, India) approved animal house.

Thirty-six adult rats were randomly assigned into 2 equal groups viz., conventional dexamethasone injection and dexamethasone microspheres. Further, in each group, 3 animals were assigned for each time point. The animals were fasted overnight, with free access to water and injected with a single dose of respective formulation (10 mg/kg body weight of dexamethasone) through the tail vein. Three rats were sacrificed at each predetermined time point viz., 0.5, 1, 3, 6, 12, and 24 h to collect the blood and organ samples [31]. Plasma was immediately separated from the blood sample by centrifugation and stored at −20 °C until analysis.

The organs viz. lungs, liver, and spleen were harvested from the animals to study the targeting efficiency [32]. Dexamethasone was extracted from the harvested organs. Briefly, the harvested organs were homogenized with methanol to extract the drug and precipitate proteins. The homogenates were centrifuged at 20,000× *g* and 4 °C. The supernatant was mixed with methyltestosterone methanol solution as the internal standard and analyzed quantitatively using the validated RP-HPLC method as mentioned previously.

Plasma concentration data were used to calculate the lung targeting ability of the dexamethasone microspheres. The tissue targeting ability of the delivery system was measured based on the relative uptake rate (*r_e_*), drug targeting efficiency (*t_e_*) and relative selectivity (*r_te_*), calculated as mentioned below [33,34]:

(1) $$r_{e}=\frac{\left(AUC{\;}_{0}^{\infty }\right)\;Test\;targeted\;delivery\;system\;}{\left(AUC{\;}_{0}^{\infty }\right)\;Conventional\;delivery\;system}$$

(2) $$t_{e}=\frac{\left(AUC{\;}_{0}^{\infty }\right)Target\;tissue}{\left(AUC{\;}_{0}^{\infty }\right)\;Non\;target\;tissue}$$

(3) $$r_{te}=\frac{(t_{e})\;dexamethasone\;microspheres}{(t_{e})\;Conventional\;dexamethasone\;injection}$$

#### 2.2.6. Induction of Lung Inflammation and In Vivo Efficacy Testing

##### LPS Induced ARDS Model in Rats

Animal studies were approved by the Institutional Animal Ethics Committee. Wistar rats (*n* = 36, weight 180–220 g) were used for the study. Rats were housed in ventilated cages and were provided food and water ad libitum. Rats were divided into four groups with nine rats in each group namely, normal control, diseased control, standard treatment, and test treatment. All the rats were anesthetized with an intraperitoneal injection of ketamine–xylazine (1:1) [35]. LPS from *Escherichia coli* 0111:B4 was dissolved in normal saline (5 mg/kg body weight), filtered aseptically through a 0.22 μm sterile syringe filter, and administered intratracheally to all the rats except the normal control group [36]. Normal control healthy group rats received normal saline intratracheally. The disease control group, standard treatment and test treatment received an intravenous injection of normal saline, marketed dexamethasone injection and microspheres of dexamethasone reconstituted in normal saline, respectively (Equivalent to dexamethasone 1 mg/kg body weight).

##### In Vivo Estimation of Inflammation Markers in Bronchoalveolar Lavage Fluid (BAL)

Animals were observed for any adverse symptoms every 30 min. from the time of intratracheal LPS instillation until the end of study. Rats were sacrificed 24 h post-dosing (*n* = 6). A tracheal cannula was inserted into the lungs and washed with 3 × 3 mL portion of chilled normal saline (4 °C). Approximately 9 mL of bronchioalveolar lavage fluid (BALF) was collected from each rat and analyzed for neutrophils (%), total protein, and pulmonary haemorrhage. Levels of lipid peroxides (Thiobarbituric acid reactive substances; TBARS), IL-1β, IL-6, and TNF-α in BALF were analyzed using ELISA kits.

##### Histopathology of Lungs

After 24 h of dosing, three randomly selected rats from each group were taken for lung histopathology studies. Lungs were collected in 10% formalin in phosphate-buffered saline. Formalized lung tissues were embedded in paraffin blocks and were then sectioned using a Leica Microtome to produce 5 μm sections. Sections were floated in a water bath, adhered to standard glass slides, and allowed to dry. Slides were then stained using standard Hematoxylin and Eosin (H&E) staining [37]. Slides were examined under the microscope and digitally photographed to document pathological changes.

#### 2.2.7. Statistical Analysis

All the experiments, unless otherwise specified, were carried out in triplicates and are represented as mean ± standard deviation. Statistical significance of the results was analyzed by one-way analysis of variance (ANOVA) at a 95% confidence limit. For assessing any significant differences between groups Newman–Keul’s test for statistical significance at 95% confidence limit was used.

## 3. Results and Discussion

Controlling inflammation is extremely critical for the effective treatment of lung injury. High oxidative stress reduces the functionality of lung surfactants eventually leading to ARDS. Further, the anatomy of the respiratory tract, presence of alveolar edema and atelectasis make the reach and effectiveness of administered drug therapies challenging. These obstacles encountered during treatment have directed the attention of researchers towards the development of targeted delivery systems achieving sufficiently high doses directly to the infection site [38]. This is of particular interest in ARDS as delivering the drug like dexamethasone selectively to the lungs will not only reduce the dose needed to achieve a therapeutic effect, but will reduce systemic side effects associated with non-target action of the drug. Dexamethasone binds the glucocorticoid receptors in the cell cytoplasm, and induces mitogen-activated protein kinase phosphatase activity. It further causes the production of annexin I thereby downregulating cytosolic phospholipase A2α expression and lowering the production of inflammatory mediators. It also downregulates NF-κB transcription reducing the production of proinflammatory cytokines like IL-1β, IL-6 and TNF-α.

Delivering dexamethasone by inhalation route becomes challenging in the case of ARDS due to severely compromised lung function. This necessitates an intravenous route of administration wherein targeting functionality can be achieved easily with the modulation of the size of the drug delivery system that will get retained in blood capillaries in lungs during mechanical filtration. The particle size of the microspheres is the main important factor as it controls the tissue location of the microspheres after intravenous administration. Previous reports have proved that microspheres with the size range of 5–25 μm have a notable lung targeting due to mechanical trapping in the fine blood capillaries of the lung [17,19,20].

We prepared dexamethasone-loaded lipopolymeric microspheres by a single emulsion technique with a lipid phase consisting of PLGA:DPPC. The lipid phase was emulsified with an aqueous phase containing vitamin E TPGS. Use of DPPC, a major component of lung surfactant in the formulation, helps to reduce the phagocytosis of microspheres by altering the cellular interactions occurring in the alveoli [39]. It has also been demonstrated that phagocytic cells have a much-reduced ability to phagocytose particles which are sterically stabilized by pendant PEG chains as compared to those stabilized by ionic charge [40,41]. In severe lung injury cases, high-localized oxidative stress inactivates the lung surfactant and increases alveolar air–liquid surface tension leading to collapse of alveolar network and atelectasis. As the microspheres degrade slowly, the DPPC will replenish the depleted surfactant reserve and will facilitate the opening up of occluded airways by virtue of its surface activity property [42] and deliver drugs into the remotest alveolar region.

Vitamin E TGPS acts as an emulsifier, solubilizer for poorly soluble drugs, permeation enhancer and also has the antioxidant potential [43]. In the alveolar milieu, it primarily acts as an antioxidant by scavenging free radicals. It also penetrates into the lipid membrane and protects the unsaturated phospholipids from free radical-mediated lipid peroxidation. It generates vitamin E in vivo, which also participates in lung surfactant biosynthesis. PLGA microspheres have been proved suitable for passive lung targeting and pulmonary therapy [44,45,46].

### 3.1. Determination of Particle Size, Distribution and Imaging Using Electron Microscopy

**Figure 1:**
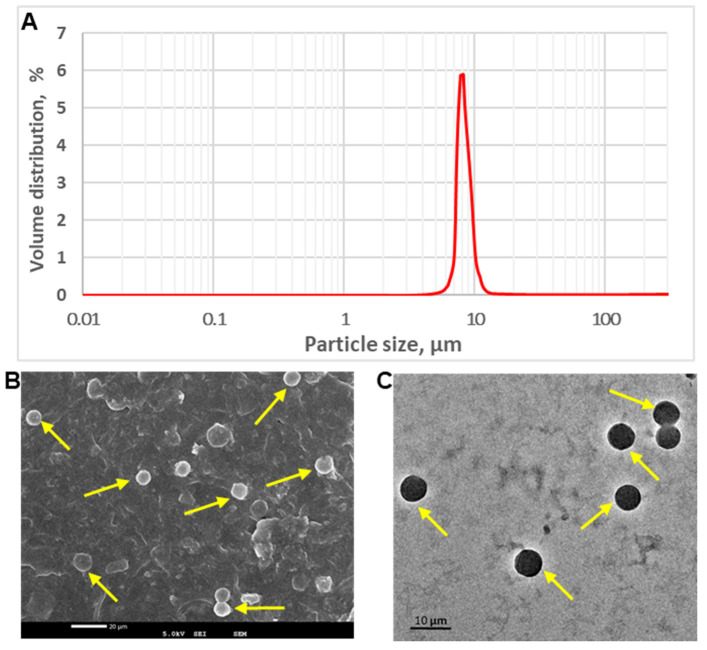
Representative graph showing particle size analysis (**A**), SEM image (**B**) and TEM image (**C**) of dexamethasone-loaded microspheres.

Dexamethasone-loaded lipopolymeric PLGA microspheres had a mean diameter of 8.83 ± 0.32 μm (*n* = 6, Figure 1A).

Polydispersity index was found to be 0.31 indicating narrow size distribution. The same is also revealed from the SEM and TEM images (Figure 1B,C) showing multiple microspheres of uniform size that were round in shape with smooth surface and absence of particle aggregation. This can be attributed to the use of an aqueous soluble surfactant; vitamin E TPGS. Overall, particle size characterization data are favorable for passive targeting of lungs. Hydrophobic PLGA microspheres were successfully prepared with high dexamethasone loading; 18.2 ± 0.9% (*n* = 3) and high encapsulation efficiency; 94.8 ± 1.8% (*n* = 3).

### 3.2. In Vitro Release of Dexamethasone

**Figure 2:**
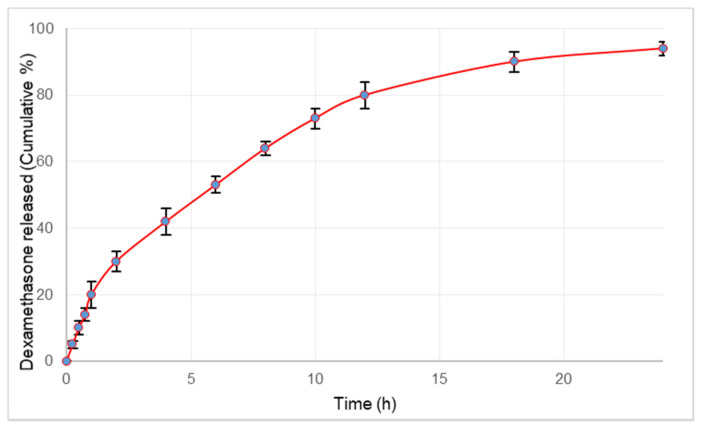
Release profile of dexamethasone from microspheres over a period of 24 h. Values plotted are the mean of 6 determinations and error bars indicate standard deviation.

The in vitro release studies showed the release of 20% dexamethasone in the initial 1 h, followed by sustained release continued up to 24 h (Figure 2). Microsphere particle size greatly affects the surface area to volume ratio, the polymer degradation rate, microsphere erosion rate and thus impacts the drug release rate from the encapsulating microsphere [45]. The size of microspheres plays a crucial role in regulating the drug release rate; increasing microsphere size results in a decreasing drug release rate and vice versa. In the present study, the lipopolymeric microspheres exhibited initial burst release of dexamethasone followed by a sustained release profile. The burst effect observed in the release can be attributed to the presence of drug particles on the surface of the microspheres. The initial burst release will ensure the loading of dexamethasone required for rapid onset of action whereas the extended release will prolong the duration of action.

### 3.3. Pharmacokinetic, Biodistribution and Targeting Efficiency

**Figure 3:**
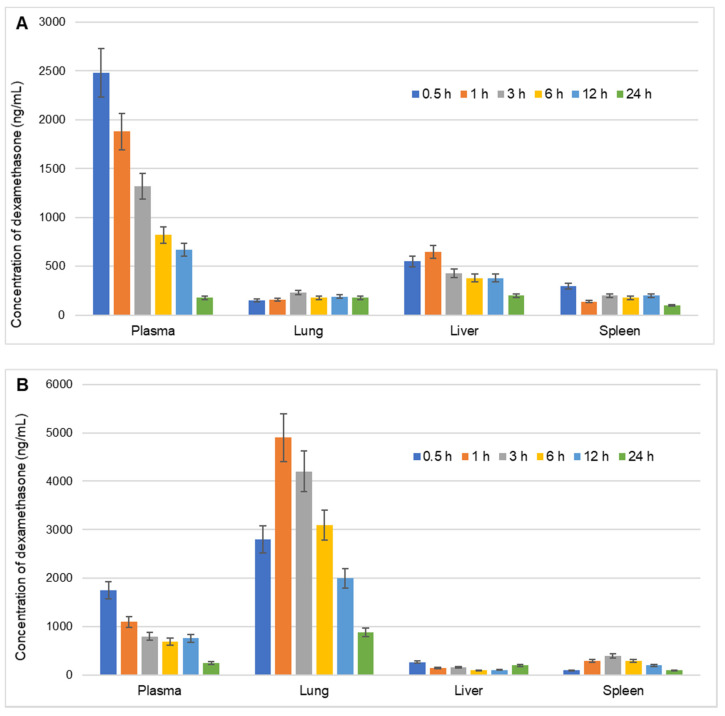
Biodistribution of dexamethasone in various organs namely, liver, lungs, and spleen (ng/g) and plasma (ng/mL) of rats at different time intervals over a period of 24 h after intravenous injection of (**A**) Conventional marketed injection of dexamethasone and (**B**) Dexamethasone-loaded lipopolymeric microspheres. Values are the mean of 3 determinations and error bars indicate standard deviation.

The biodistribution profile of the dexamethasone-loaded PLGA microspheres was compared with the conventional dexamethasone injection (Figure 3) and various derived parameters are listed in Table 1.

A comparative pharmacokinetic profile of the dexamethasone indicates higher lung accumulation when administered as lipopolymeric PLGA microspheres (Figure 3A,B). To have an in-depth understanding of the organ targeting ability of the dexamethasone microspheres, *r_e_, t_e_* and *r_te_* were calculated. The *r_e_* implies time-averaged relative drug exposure to an organ. *R_e_* for lungs, was found to be 13.98 when dexamethasone was administered as microspheres. A *r_e_* > 1 for a tissue or organ indicates a greater exposure of that tissue or organ to the drug following the administration of test formulation [47]. Thus, significantly higher *r_e_* for lungs as compared to the other organs namely spleen and liver (1.34 and 0.45, respectively) and plasma as well (0.84), indicates the targeting ability of the microspheres to the lungs. Another parameter, *t_e_* signifies selectivity i.e., drug targeting efficiency of a delivery system against a given non-target tissue [48]. In the present study, *t_e_* for each organ was calculated against plasma levels. The *t_e_* of dexamethasone-loaded PLGA microspheres was found to be 3.88 in comparison to conventional dexamethasone injection (*t_e_* = 0.23) demonstrating preferential accumulation of the dexamethasone microsphere formulation in lung tissue as compared to the conventional injection formulation. A lower amount of dexamethasone microsphere in liver (*t_e_* = 0.19) is also indicative of the reduced elimination and higher localized accumulation of dexamethasone microspheres. The lower selectivity of dexamethasone-loaded PLGA microspheres for the liver and spleen, will ensure that the formulation is less likely to be rapidly eliminated from the body. This will help to maintain the optimum localized drug concentration in the lungs for a longer duration.

To draw a more comprehensive comparative targeting ability of microsphere formulation another parameter, *r_te_,* was calculated. The parameter *r_te_* expresses the comparative drug targeting ability of two delivery systems (herein, dexamethasone microsphere vs. conventional dexamethasone injection) to modulate the target: non-target tissue distribution of a drug [48]. Comparison of *r_te_* indicates the differential selectivity and uptake of the molecule when presented via different drug delivery technology. The *r_te_* values for lung, spleen, and liver were 16.56, 0.53, and 1.58, respectively, indicative of lungs as the preferred target for the developed microspheres.

The comparative evaluation of the targeting ability of the dexamethasone microsphere against conventional dexamethasone injection shows that the dexamethasone-loaded lipopolymeric microspheres significantly increases the drug accumulation in the lungs along with lower drug distribution to the other non-target tissues. This will be desirable to avoid the possible potential toxic effects of dexamethasone on organs of the body. The results indicated that the lipopolymeric PLGA microspheres could preferentially deliver dexamethasone specifically to the lung after intravenous injection (Figure 3).

### 3.4. In vivo Efficacy in LPS Induced ARDS Model

To verify the therapeutic efficacy of dexamethasone-loaded PLGA microspheres and dexamethasone injection (marketed product) were injected intravenously in ARDS-bearing rat models following the protocol described in the methods section. LPS is known to elicit an acute inflammation in lungs progressing to lung injury and has been used to model for ARDS and in animal studies [49]. In the present study, intratracheally instilled LPS from *Escherichia coli* O111:B4 was used to elicit an inflammatory response in rat lungs.

**Figure 4:**
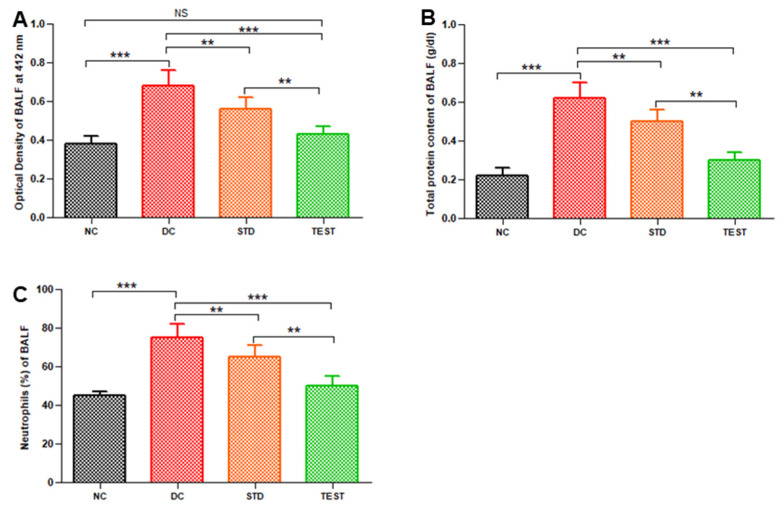
Pulmonary haemorrhage in terms of optical density of BALF (**A**), total protein (**B**), and neutrophil (**C**) in BALF from various groups namely, Normal Control (NC), Disease Control (DC), standard treatment of conventional dexamethasone injection (STD), and test formulation treatment with dexamethasone microspheres (TEST) (*n* = 6). ** and *** represent significant differences at *p* < 0.01 and *p* < 0.001, respectively, by Newman–Keuls analysis following ANOVA at 95% confidence limit. NS = Non-significant.

The changes in optical density (an indicator of haemorrhage), total proteins, and neutrophils in BALF were determined to validate the therapeutic efficacy of the developed formulation. Notably, dexamethasone-loaded PLGA microspheres significantly reduced the haemorrhage as observed from the reduced optical density of BALF (Figure 4A) at a dose of 1 mg/kg.

The number of neutrophils present in BALF was represented as a percentage of the total leucocyte population counted in the BALF samples (Figure 4C). BALF from the diseased group had a significantly high percentage of neutrophil accumulation (76 ± 5%) in comparison to normal control (42 ± 3%). BALF of standard treatment and test treated group contained 64 ± 5% and 50 ± 4% neutrophils, respectively. Marked reduction in neutrophil recruitment is seen in microspheres treated group (ANOVA, *p* < 0.001).

**Figure 5:**
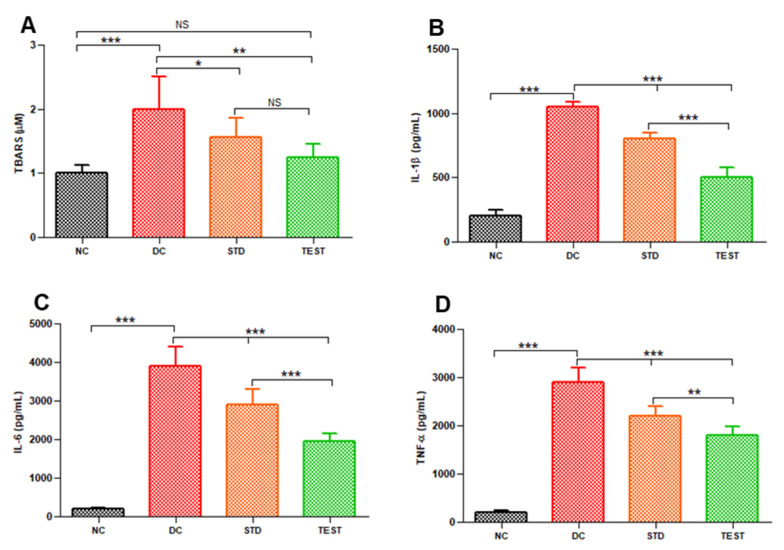
Levels of lipid peroxidation and various inflammatory markers in BALF from various groups namely, Normal Control (NC), Disease Control (DC), standard treatment with conventional dexamethasone injection (STD) and test formulation treatment with dexamethasone microspheres (TEST) (*n* = 6) (**A**) TBARS, (**B**) IL-1β, (**C**) IL-6, and (**D**) TNF-α. *, **, and *** represents significant difference at *p* < 0.05 and *p* < 0.01 and *p <* 0.001, respectively, by Newman–Keuls analysis following ANOVA at 95% confidence limit. NS = Not significant.

Oxidative stress was measured as TBARS. BALF from the diseased control group has significantly higher TBARS of 1.9 ± 0.5 μM in comparison to 1.00 ± 0.12 μM for the normal control group (*p* < 0.001, Figure 5A). The high capillary permeability in the diseased control group results in higher recruitment and activation of neutrophils in the alveoli that further increases oxidative stress and causes lung surfactant dysfunction. Neutrophil infiltration in lungs was reduced in dexamethasone-treated groups along with associated lower TBARS values in BALF indicating the effect of dexamethasone towards reducing the inflammation and related oxidative stress. However, the effect was more significant in the microspheres treated group (TBARS 1.25 ± 0.5 μM, *p <* 0.01) compared to conventional injection (1.56 ± 0.3 μM, *p* < 0.05).

We also quantified the levels of cytokines namely IL-1β, IL-6, and TNF-α levels in BALF (Figure 5B–D).

It can be seen from the above figure that between the dexamethasone conventional formulation and the developed microspheres, the latter was most effective in reducing the expression of inflammatory cytokines and thus can potentially help to reduce inflammation in ARDS. This might be because of the synergistic effect of the combination of Vit E TPGS and dexamethasone. Vitamin E TPGS generates Vitamin E in vivo that acts as a free radical scavenger and reduces the load of local reactive oxygen species in the lungs. LPS and the generated proinflammatory cytokine IL-1β further stimulate the production of macrophage inflammatory proteins. Vitamin E reduces these inflammatory proteins. Compared to conventional dexamethasone formulation, lipopolymeric microspheres were significantly more capable of controlling the release of inflammatory mediators. The dexamethasone microspheres formulated significantly lowered the levels of inflammatory mediators (Figure 5B–D, *p* < 0.005) in comparison to the conventional treatment with dexamethasone drug alone.

### 3.5. Histopathology Studies

**Figure 6:**
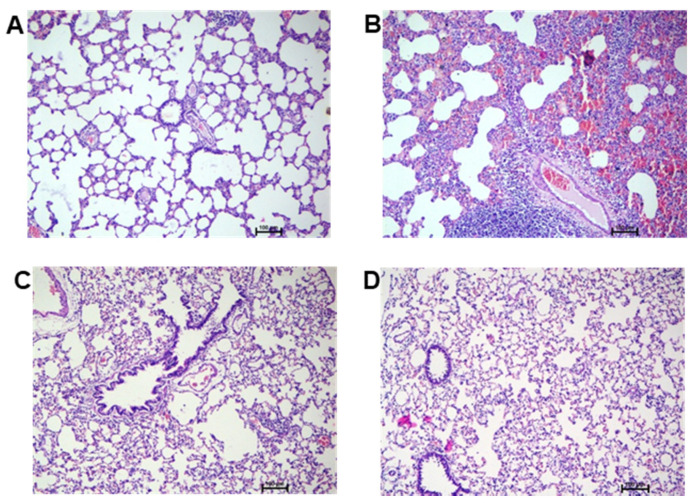
Haematoxylin and eosin (H,E)-stained representative rat lung tissue histopathology images of (**A**) normal control, (**B**) diseased control, (**C**) standard treatment with conventional dexamethasone injection, and (**D**) developed test formulation treatment.

Histologic evaluation is performed on hematoxylin and eosin-stained lung sections of rats. In the control group (Figure 6A), the normal pulmonary structures, such as the alveolar septa, alveolar lumen, and capillary, are well preserved. Inflammatory cell infiltration and pneumatic changes were not observed. In the diseased control group (Figure 6B), focal infiltration of mononuclear cells in the lung parenchyma/alveoli was observed. Edematous changes with exudate in the interstitial space and the alveolar lumen were also detected. Pneumonic changes with the consolidation of alveolar parenchyma with diffuse haemorrhagic feature and acute reactive inflammatory changes in the lung tissue/parenchyma with a moderate disease score are clearly evident. In the standard treatment group (Figure 6C), the therapeutic effect of dexamethasone is observed. However, pathological changes were still visible with a mild disease score with inflammatory and haemorrhagic features in the tissue. In the test formulation group (Figure 6D), there was a significant recovery with respect to histological features, indicating restoration of alveoli structure to normal condition. Additionally, there was an absence of inflammatory cellular features and pneumatic changes in the lung parenchyma. No significant abnormalities were observed when compared with disease control (Figure 6B) and standard drug treatment (Figure 6C). The results suggest the potential targeting of the dexamethasone to the lung through lipopolymeric microspheres.

## 4. Conclusions

ARDS, a catastrophic illness of multifactorial etiology, characterized by a rapid upsurge in inflammatory cytokines [50], leads to hypoxemic respiratory failure. There is no specific treatment for ARDS at this time. The currently available strategies focus on supporting the patient by intravenous medication while the lungs heal. Administration of dexamethasone could reduce morbidity and mortality in patients with established moderate-to-severe ARDS. However, chronic use of conventional dexamethasone might lead to severe side effects including organ dysfunction. Hence, lipopolymeric PLGA microspheres loaded with dexamethasone with passive lung targeting functionality were developed successfully. Spherical and uniform size dispersed microspheres showed higher targeted efficiency and selectivity for lung tissue compared to other organs. Treatment in the ARDS animal model clearly showed substantial inhibitions of the protein infiltration and neutrophil accumulation in the lungs. Histopathology showed no pulmonary haemorrhage or inflammation upon treatment with the dexamethasone-loaded microspheres. The results suggest that the prepared dexamethasone-loaded PLGA microspheres can selectively be delivered to lung tissue for effective therapeutic response.

Microspheres offer a flexible and versatile scalable technology that can be adapted to a variety of drugs by modulating the process parameters to achieve requisite pharmaceutical characteristics which can be easily correlated to pharmacological responses. The result of this study would guide formulation strategies to achieve commercial success with the optimum utilization of the therapeutic response by modulating delivery vehicles. Such microspheres can also serve as a platform technology to deliver conventionally used drugs for systemic administration, steroidal as well as non-steroidal anti-inflammatory drugs to the lungs without causing extensive systemic side effects. In vivo efficacy studies in different lung injury models using different injury-causing agents will be highly crucial in establishing the true potential of such drug delivery systems in the treatment of acute lung inflammatory conditions.

## Figures and Tables

**Table 1:** Comparative biodistribution of dexamethasone in plasma, lung, liver and spleen after intravenous of conventional dexamethasone injection and developed dexamethasone microspheres.

Plasma/Organ	$r_{e}$	Conventional Dexamethasone Injection *t_e_*	Dexamethasone Microspheres *t_e_*	$r_{te}$
Plasma	0.84	-	-	-
Lung	13.98	0.23	3.88	16.56
Liver	0.45	0.36	0.19	0.53
Spleen	1.34	0.23	0.36	1.58

## Data Availability

All the relevant data is included in the manuscript.
